# Short-term senolytic treatment: a paradigm to promote fracture repair during aging

**DOI:** 10.1172/JCI158871

**Published:** 2022-04-15

**Authors:** Isabel Beerman, Nathan Basisty, Rafael de Cabo

**Affiliations:** Translational Gerontology Branch, National Institute on Aging (NIA), NIH, Baltimore, Maryland, USA.

## Abstract

Increased age is blamed for a wide range of bone physiological changes, and although the underlying mechanisms affecting the decreased capacity for fracture healing are not fully understood, they are clearly linked to changes at the cellular level. Recent evidence suggests potential roles of senescent cells in response to most tissue injuries, including bone fractures. In this issue of the *JCI*, Liu, Zhang, and co-authors showed that a senolytic drug cocktail cleared senescent cells from the callus and improved bone fracture repair in aged mice. Understanding how senescent cells emerge at fracture sites and how their timely removal improves fracture healing should provide insights for effective therapeutic approaches in old age.

## Senescence and TGF-β1 as targets for bone fractures

Oscar Wilde once said, “*With age comes wisdom.*” Sometimes and often, age does not come alone. Aging is the highest risk factor for most diseases and conditions that limit normal organismal function. The skeletal system is particularly affected by increasing age, raising the impact of osteoporosis and osteoarthritis and leading to higher rates of bone fractures with delayed healing that are associated with temporal incapacitation and, ultimately, increased morbidity and mortality (1). Several fundamental aging processes likely contribute to the decline in fracture healing potential, including inflammaging, loss of stem cell potential, and, notably, increased levels of senescence in the callous, a cartilaginous material that bridges the bone fracture during repair (2). In this issue of the *JCI*, Xing’s team used a short-term, intermittent treatment with a senolytic drug cocktail, dasatinib and quercetin (D+Q), to clear senescent cells from the callus and improve bone fracture repair in aged mice (3). This result was phenocopied by inhibiting TGF-β1 signaling, a component of the senescence-associated secretory phenotype (SASP) that contributes to age-related pathologies. These promising results suggest that targeting basic mechanisms of aging, such as cellular senescence, may be therapeutically exploited for enhanced bone fracture repair in the older population. The findings also raise important questions about the effects of aging and optimal treatment regimens, including the timing of initiation, dosages, and duration.

## Timing and dosages in a senolytic treatment paradigm

Liu, Zhang, and colleagues (3) demonstrated that intermittent, short-term administration of the senolytic drug cocktail D+Q was sufficient to decrease the markers of senescence after trauma and improve fracture healing in older male and female mice, but not in their younger counterparts. The results suggest that senolytic treatment targets a fundamental aging mechanism that can be potentially exploited for treatments tailored to older individuals. During aging, a therapeutic window may exist, in which short-term senolytic treatments could boost the organism’s bone repair potential. However, if senescent cells are present at the site of repair, regardless of the age of the individual, defining the window of treatment opportunity is important to maximize therapeutic benefit. Experiments should evaluate the effects of senolytic or senomorphic (SASP-modulating) treatments at various ages to identify the limits of therapeutic benefit and determine whether the duration of treatment could be tailored to the person’s age. Given interindividual variations associated with aging, it is of strong interest to identify systemic markers that could be used to indicate potentially senolytic-responsive individuals. For example, circulating SASP factors and cytokines, such as TGF-β1, may serve as markers to indicate a potential treatment window. The development of senescence biomarker signatures is an area of highly active research that will surely aid in this endeavor (4–8).

Regardless of the optimal therapeutic window for treatment, Liu, Zhang, and colleagues (3) described a tailored protocol that improved fracture healing in old mice, while mitigating the side effects associated with senolytic drugs. Although future studies need to determine the optimal dosage and duration, a short, intermittent dosing regimen offers promising clinical translation with fewer negative off-target effects that sustained administration of current senolytic drugs carry.

## A need for senescence-specific markers

Senescent cells are functionally heterogeneous, depending on the cell type and the stimuli. They are important mediators of regeneration in some contexts, while detrimental in others (9, 10). Thus, proper timing and/or duration in the delivery of senotherapeutics may limit potential off-target effects, such as injury to other cell types that share markers with senescent cells. For instance, D+Q treatment improves skeletal muscle regeneration and repair after injury in aged mice, whereas it is ineffective in young mice (11). One week after muscle injury, immune-related senescence-associated cells emerge in the damaged environment, as indicated by senescence-associated β-gal (SA–β-gal), and are depleted in response to D+Q treatment (11).

Macrophages are essential for bone development and the various fractured bone healing stages: inflammatory, reparative, and remodeling. Excessive depletion of senescent cells with senolytics may have negative consequences. Indeed, macrophages can transiently express high levels of p16^INK4a^ and SA–β-gal activity, and systemic depletion of cells with senescence markers may promote adverse macrophage loss at the callus (12, 13). Thus, there is a need for markers that define and target growth-arrested cells as opposed to cells temporarily expressing senescence markers. Similarly, administration of a senolytic drug for extensive periods of time may deplete the types of cells required for tissue remodeling. Genetic depletion of macrophages and other mononuclear phagocytes in a CSF-1–knockout mouse model results in osteoporosis and blunted bone development (14), while macrophage depletion with clodronate liposomes prior to bone surgical fracture impairs bone healing (15).

## Alternative mechanisms of bone repair

The importance of immune cells in bone fracture healing is highlighted by the fact that macrophages secrete several factors, including TGF-β1, to the area of injury. TGF-β1 and other inflammatory signals trigger the recruitment of mesenchymal stem cells (MSCs) (16) that have the potential to differentiate into other types of cells. Low levels of the TGF-β family members drive osteoclast differentiation, whereas elevated concentrations inhibit osteoclast formation (17). In the aged mice, in which the levels of TGF-β1 are already high, Liu, Zhang, and co-authors (3) showed that reducing TGF-β1 levels improved bone marrow repair, perhaps by promoting osteoblast differentiation. These findings showed that a short period of treatment after injury was sufficient to improve bone repair in the aged mice and that the timing for TGF-β1 inhibition was critical for the differentiation potential of MSCs. These results indicate that the bone remodeling benefits of TGF-β1 may only be borne out in environments with higher starting levels of this growth factor.

Reports of direct, positive benefits of D+Q treatment in MSCs (18) and chondrogenic progenitor cells (19) complement studies using other senolytic agents, such as ABT263, navitoclax, catechins, and a FOXO4-DRI peptide, in other tissue-specific stem cells and progenitor cells (20). Interestingly, the targets of these senolytics include critical pathways for the function and maintenance of many adult stem cells. A more comprehensive understanding of the importance of the dosage and timing of the drugs and their potential impact on other tissues is warranted. In the hematopoietic system, senolytics, including inhibitors of antiapoptotic proteins, are currently being used in cancer treatment, guided mostly by the fact that cancer stem cells rely more on B cell lymphoma 2 (BCL-2) pathways for survival than do normal hematopoietic stem cells (21). While toxic to malignant stem cells, administration of selective BCL inhibitors may have lesser effects on native stem cells. Studies aimed at comparing dose-response curves of senolytics in tissue-specific stem cells versus in vivo senescent cells remain a challenge, given our limitations in defining and isolating rare, heterogeneous senescent cell populations in vivo. 

## Conclusion

The role, physiology, and pathobiology of senescent cells is a blooming area of scientific interest across research fields. Multiple studies point to the accumulation of senescent cells as a driving force of age-related tissue deterioration and the beneficial effects of their removal. So far, most of the proposed mechanisms and modulators have other functions outside of senescence, thus confounding the true contributions of each response during the tissue repair process and aging. Can we truly distinguish between good and bad senescent cells, and can we selectively target them to prevent off-target effects? Without a doubt, there are a multitude of beneficial roles for senescent cells during development, immune responses, and tissue repair processes, highlighting the complexity of the roles of these cells and how little we truly understand their origin, functions, and biology. 

The promising results from multiple senolytic treatments and the data showing robust responses to short-term dosing after injury in specific tissues during aging both contribute to the exciting potential of translating these treatments to humans. However, it will be critical to understand the global effects of the drugs and to unravel the heterogeneous response of senescent cells following administration of senolytics. An additional focus toward defining the specific roles of the different subsets of senescent cells that reside in an organism or tissue, or senotype, will be critical to understanding the global effects of senolytic treatments. Toward this end, the NIA has established a common fund’s Cellular Senescence Network (SenNet) Program to generate comprehensive atlases of senescent cells that arise in both humans and mice during aging and under healthy and diseased states across multiple tissues. These databases should shed light on the definition of the different phenotypes of senescent cells in the context of time, how each particular phenotype is associated with their function, and whether a given phenotype is beneficial or detrimental for tissue repair and function. Importantly, these studies will provide highly senotype-specific biomarkers that will help identify the therapeutic window for senolytic interventions and guide the dosage, timing, and duration of senolytic treatments in the aging population.

## Author contributions

The order of the authors was determined alphabetically by first initials.

## Version 1 04/15/2022

Electronic publication
